# Tribological Properties of a Sliding Joint with an a-C:H:W Coating under Lubrication Conditions with PAO8 Oil and the Addition of 2% MoS_2_ Nanoparticles

**DOI:** 10.3390/ma17040870

**Published:** 2024-02-13

**Authors:** Krystian Hadło, Janusz Lubas, Wojciech Szczypinski-Sala, Agnieszka Tomala, Dariusz Konieczny

**Affiliations:** 1 Faculty of Mechanical Engineering and Aeronautics, Rzeszow University of Technology, Powstańców Warszawy 8 Av., 35-959 Rzeszow, Poland; krystian.hadlo@gmail.com (K.H.); dkoniecz@prz.edu.pl (D.K.); 2 Faculty of Mechanical Engineering, Cracow University of Technology, 37 Jana Pawła II Av., 31-864 Krakow, Poland; wojciech.szczypinski-sala@pk.edu.pl; 3 Faculty of Materials Engineering and Physics, Cracow University of Technology, 37 Jana Pawła II Av., 31-864 Krakow, Poland; agnieszka.tomala@pk.edu.pl

**Keywords:** coating, wear, friction, surface layer, DLC, engine oil, MoS_2_

## Abstract

One of the promising methods for improving the durability and reliability of friction joints in combustion engines is the use of thin and hard coatings, including coatings based on amorphous DLC. The a-C:H:W coating was produced using the commercial PVD method. The tested tribological joints were made of AISI 4337 steel and SAE-48 bearing alloy (conformal contact) and AISI 4337 steel and valve shims (non-conformal contact). The contact area was lubricated with SAE 5W40 engine oil and PAO8 oil + 2 wt.% MoS_2_ nanoparticles. The objective of this work is to explore the influence of PAO8 + MoS_2_ on the tribological properties of a sliding joint with an a-C:H:W coating and the change in the properties of the oils. In the conformal contact, the lubrication of the a-C:H:W coating with PAO8 + MoS_2_ caused a significant increase in the friction resistance (than in) as compared to the joints with a quenching and tempering surface layer and lubricated SAE 5W40, while in the non-conformal contact, the lubrication of the a-C:H:W coating with PAO8 + MoS_2_ caused a decrease in the friction resistance and temperature of the contact area. The joints with the a-C:H:W coating were characterized by higher wear of the SAE-48 bearing alloy, as compared to the joints with the surface layer without coating (lubricated with SAE 5W40 oil—11-fold increase, PAO8 + MoS_2_—46-fold increase). The wear of valve shims with the a-C:H:W coating was significantly lower as compared to the wear of the commercial version of the valve shims (the difference between joints lubricated with SAE 5W40 oil and PAO8 + MoS_2_ was 12%, 36% and 29% for unit pressures of 10, 15 and 20 MPa). Lubrication of the a-C:H:W coating with PAO8 oil + MoS_2_ protected the sliding joints against seizing in non-conformal contact.

## 1. Introduction

In a lubricated mechanical machine, materials with identical mechanical properties are usually not used in the entire cross-section of the elements constituting mechanical pairs. Important tribological properties depend on the properties and structure of the surface layers of the elements of mechanical pairs, the lubrication conditions of lubricants and the lubrication methods [1,2,3]. The development of technology has introduced new fabrication methods of surface layers and the improvement of existing solutions to increase anti-wear and anti-seize properties [4]. Surface layers and coatings are required to combine properties such as hardness, shear resistance, cracking resistance and good adhesion to the substrate [5,6,7,8]. The use of PVD (Physical Vapor Deposition) and CVD (Chemical Vapor Deposition) methods in forming coatings is systematically growing [9,10]. These methods enabled us to obtain coatings with different characteristics [11,12,13]. As was previously described (Baptistai et al. [14,15]), hard coatings can serve their purpose in different conditions, and also in the presence of abrasive particles. They can be applied on any surface particularly exposed to wear [16,17]. A significant area of use for hard coatings is machining tools [18]. As described by Silva et al. [19], the application of hard coatings significantly reduces abrasion. As the authors stated, in a case of using coatings on the surface of molds for injection: “the wear resistance of the mold was increased by a factor of 25 and 58, by the TiAlSiN and CrN/CrCN/DLC coatings, respectively, over the uncoated mold steel”. In each area of application, it is necessary to search for new coating compositions with even better qualities [19]. The coatings formed by PVD and CVD methods allow the formation of single- or multilayer thin anti-wear and protective coatings, which are an alternative or a supplement to the classical methods of producing surface layers [20,21]. At the NASA (The National Aeronautics and Space Administration) Glenn Research Centre, PVD coatings spectacularly improve bearing and gear performance in transient conditions and in emergency cases [8]. Generally, very thin coatings are obtained using this method [22].

Coatings based on amorphous DLC (diamond-like carbon) can be divided into coatings with the structure of anhydrous amorphous carbon and hydrogenated amorphous carbon without any dopes or with metal and non-metal dopes [23,24,25,26,27]. DLC coatings have several advantages: low coefficient of friction, high hardness, high wear resistance, low coefficient and good adhesion [28,29]. They are also chemically inert and can work under dry friction conditions. Another advantage of DLC coatings is their thickness, which allows them to be shaped without the need for additional machining of the parts to maintain the dimensions and tolerances of the mechanical pairs [25,26]. These issues are also described in a number of publications [30,31]. These are coatings with different characteristics, as pointed out by Casais et al. [32]: “CrN/CrCN/DLC multilayered coatings presented a better overall wear behavior, whereas B_4_C coating showed a good wear behavior regarding the load”. Among the carbon coatings characterized by favorable tribological properties, there are coatings doped with tungsten, i.e., a-C:H:W (W-incorporated hydrogenated DLC) coatings. a-C:H:W coatings contain metals (a-C:H:Me), and basic bench tests indicate their usefulness in highly loaded lubricated sliding joints [33,34].

Transition Metal Dichalcogenide (TMD) nanoparticles such as MoS_2_ and WS_2_ are some of the most utilized solid lubricants with a wide range of applications. TMD applied in the form of thin layers causes strong friction reduction properties [35]. The use of TMD nanoparticles in standard oils improves their tribological properties (anti-wear, anti-seizure), and friction-reducing properties between moving mechanical components [36,37,38]. Molybdenum disulfide is a substance with a layered structure, resistant to high temperatures, and is characterized by good adhesion to the substrate. Studies have shown that the addition of nanoparticles (n-MoS_2_) can improve the tribological properties of base oils [39,40,41]. The addition of MoS_2_ nanotubes to the base PAO oil significantly decreases the friction coefficient and reduces wear under boundary lubrication conditions [42,43]. Mineral oils with nano-MoS_2_ also cause a reduction of wear and friction coefficients in tribological pairs; moreover, the wear mechanism of MoS_2_ indicates that it can be used as an anti-wear additive in sliding joint [44].

Due to the design, load and lubrication conditions and material configuration, it is not possible to unambiguously describe the tribological processes occurring in sliding joints with elements with a-C:H:W coating. For this reason, research was undertaken in the present work, including an analysis of the friction conditions in joints made of materials occurring in lubricated real joints in internal combustion engines, taking into consideration both conformal and non-conformal contact. An important part of this work is the analysis of changes in the anti-seizure properties of oils after working in sliding joints containing elements with a-C:H:W coating under conditions of limited lubrication.

## 2. Materials and Methods

**Figure 1:**
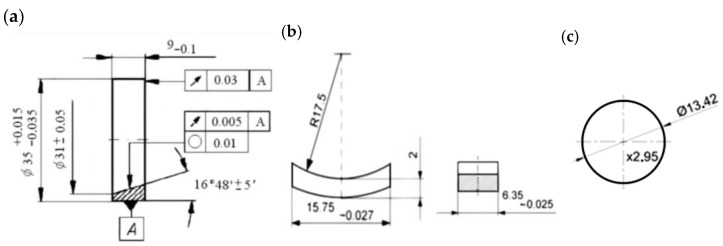
Elements of the tested joint: (**a**) ring specimen, (**b**) counterpart with SAE-48 bearing alloy (conformal contact), (**c**) counterpart—valve shims (non-conformal contact).

**Figure 2:**
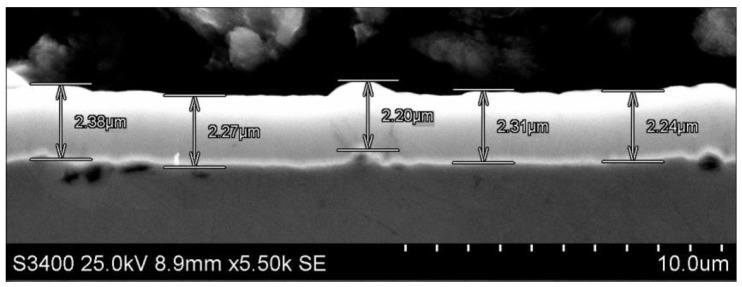
SEM cross—section a-C:H:W coating.

Low-alloy AISI 4337 steel is used on elements in combustion engines (Table 1). The ring samples with AISI 4337 steel were quenched and tempered (38 HRC ± 2) (Figure 1). The a-C:H:W coating was fabricated using the commercial PVD method (Oerlikon Balzers Coating Poland, Polkowice, Poland). The thickness of the coating was min. 2 μm, with a hardness of 10–15 GPa (Figure 2). The joints with conformal contact contained a counterpart with the journal bearing SAE-48 alloy (Figure 1) and the joints with non-conformal contact contained commercially produced valve shins (hardening 61 ÷ 63 HRC) (Figure 1).

**Figure 3:**
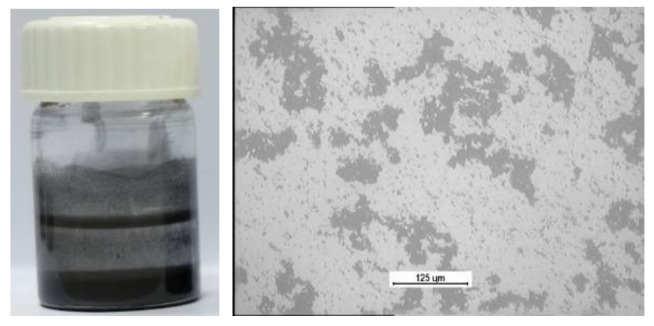
Photo of dispersed nanoparticles 50 h after homogenization.

The friction area during the tests was lubricated with SAE 5W40 oil and PAO8 base oil + MoS_2_ (2 wt.% concentration of MoS_2_ nanoparticles (NPs) (Table 2). The nanoparticles (NPs) used to lubricate the contact area of joints were synthesized from Mo8S2I8 nanowires (diameter of the NPs was 100–150 nm) [45]. The PAO8 base oil and nanoparticles NPs were mixed using an ultrasonic processor, VC 505 Sonics & Materials Sonics & Materials, Newtown, CT, USA. The suspension stability of PAO8 + MoS_2_ was 10 h until complete precipitation of MoS_2_ nanoparticles (estimated using an optical camera). The analysis with optical microscopy visualized the dispersity of the NPs: it can be observed that large agglomerates were formed in the mixed PAO8 + MoS_2_ NPs (Figure 3).

**Figure 4:**
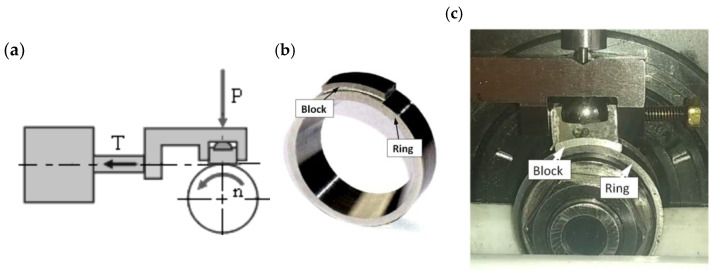
Simplified scheme of the block-on-ring tester (**a**), the sliding joints—ring and block (**b**) and the joint in machine T-05 (**c**); n—rotational speed, P—load, T—resistive force.

The comparative tests were carried out on the block-on-ring tester (Figure 4) [46]. Experiments were carried out in accordance with the ASTM D 2981 [47], ASTM D 3704, ASTM G 77 and ASTM D 2714 standards. The tests included a cooperation pair under unit pressures of 5, 10, 15 and 20 MPa in conformal contact and under forces of 1800, 2400 and 3000 N in non-conformal contact. In the friction pair during startup tests, the ring was accelerated from 0 to 1 m/s over 15 s, during wear tests the ring had a rotational speed of 100 rpm, and the friction test continued for 1000 s. The measured parameters during the friction test were the friction coefficient and the temperature in the friction area. The ring specimens were embedded in oil.

The scuffing test of the oil was performed on a four-ball testing machine (diameter of the balls—12.7 mm, hardness—62 HRC ± 2, material—100Cr6 steel) [46]. The tests were performed using the dynamic method, in which the load on the friction joint increased linearly from 0 to 7200 N for 18 s (409 N/s). The test result was taken as the arithmetic average of three repetitions that did not differ from their arithmetic average by more than 10%. The scuffing test was carried out for the unused oils and then for those used in non-conformal contact in friction pairs with commercial valve shims and with valve shims with a-C:H:W coating after 5400 s of test duration.

## 3. Results

**Figure 5:**
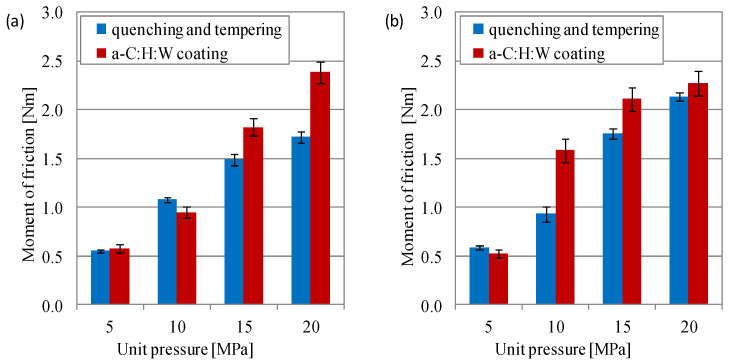
Moment of friction during start-up in joints with conformal contact: (**a**) SAE 5W40, (**b**) PAO8 + MoS_2_.

The maximum moment of friction was lower in the joints with a quenching and tempering surface layer than in the joints with the a-C:H:W coating. Under the load of 10 MPa (lubricated with SAE 5W40 oil) and under the load of 5 MPa (lubricated with PAO8 + MoS_2_), the measured values were higher for joints with a quenching and tempering surface layer than for joints with the coating. The sliding joints lubricated with PAO8 + MoS_2_ had higher moments of friction compared to those lubricated with the SAE 5W40 oil, especially under higher unit pressure. The same was observed for joints with a quenching and tempering surface layer at a unit pressure of 15–20 MPa and joints with coating at a unit pressure of 10–15 MPa. In the joint lubricated with the SAE 5W40 oil at a load of 20 MPa and in the one lubricated with the SAE 5W40 oil, the moment of friction was 45% higher than in the joints with a coated ring, as compared to the joint with a quenching and tempering ring. And in the joints with a-C:H:W coating at a load of 10 MPa and lubricated with PAO8 + MoS_2_, the moment of friction was about 63% higher than in the joints with a quenching and tempering surface layer (Figure 5).

**Figure 6:**
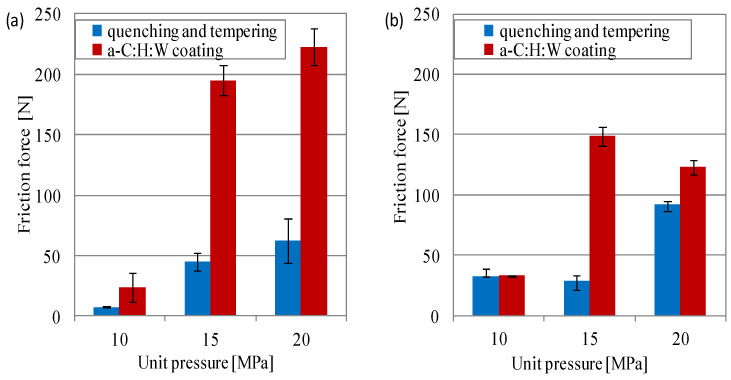
Friction force vs. unit pressure at the end of the test in joints with conformal contact: (**a**) SAE 5W40, (**b**) PAO8 + MoS_2_.

**Figure 7:**
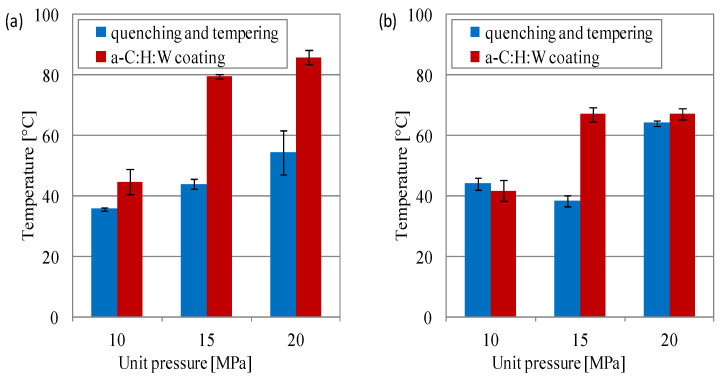
Temperature in contact area vs. unit pressure at the end of the test in joints with conformal contact: (**a**) SAE 5W40, (**b**) PAO8 + MoS_2_.

The friction force and temperature measured in the area in conformal contact allowed us to determine the working conditions of the joints (Figure 6 and Figure 7). When lubricated with SAE 5W40 oil, the friction force in the joints with a coating was higher than in the case of the joints with a quenching and tempering surface layer. Under these friction conditions, there was a proportional increase in the friction force with increased unit pressure (Figure 6a). The sliding joints lubricated with PAO8 + MoS_2_ also showed a higher friction force in the joints with a coating than in the joints with a quenching and tempering surface layer, but only at higher unit pressures (15–20 MPa). At a pressure of 5 MPa, the friction force was similar (Figure 6b). This is in line with the observation of Tomala et al. in [48], where the strong chemical reactivity between the a-C:H coating and the MoS_2_ NP-based additives caused the intensive wear of coating at high contact pressures. The use of PAO8 + MoS_2_ for the lubrication of the sliding joints caused a reduction in friction force, especially in the joints with coating under high loads, while an increase was seen in joints with a quenching and tempering layer at a load of 20 MPa. The greatest difference in the friction force occurred during lubrication with the SAE 5W40 oil. Under these lubrication conditions, the friction force was about 3 times lower in joints with a quenching and tempering surface layer at 10 MPa load, 4.5 times lower at 15 MPa, and 3.5 times lower at 20 MPa, as compared to joints with the a-C:H:W coating. In the joints lubricated with PAO8 + MoS_2_, at loads of 15 MPa and 20 MPa, the friction force was lower, too. In the joints with a quenching and tempering surface layer, the friction force was five times lower as compared to the joints with a coating (Figure 6).

The measured temperature in the contact area was higher in the sliding joints with the a-C:H:W coating than in the joints with a quenching and tempering surface layer (Figure 7). When lubricated with the SAE 5W40 oil, it was observed that the temperature differences were much larger and amounted to about 25%, 44% and 36%, respectively, for loads of 10–20 MPa (Figure 7a). When PAO8 + MoS_2_ was used, the temperature differences were small, especially at loads of 10 and 20 MPa. While at 10 MPa, the temperature was lower in the joints with a coating than in those with a quenching and tempering surface layer by approximately 7%, at 20 MPa, it was approximately 4% higher (Figure 7b).

**Figure 8:**
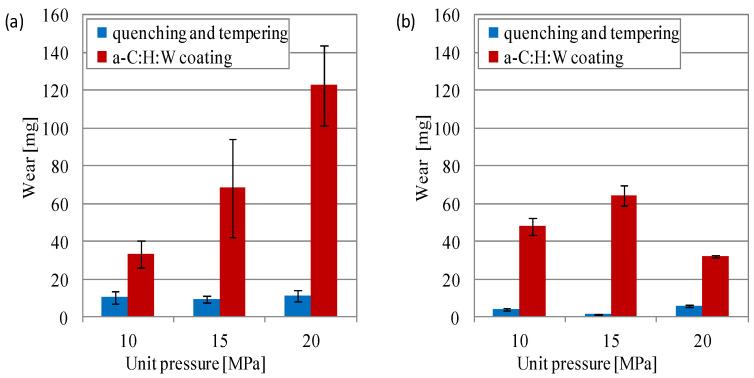
Mass wear of the SAE-48 alloy during lubricated with: (**a**) 5W40, (**b**) PAO8 + MoS_2_.

The analysis of wear of the elements of sliding joints showed strong wear processes in the joints with the SAE-48 alloy. The mass wear of the SAE-48 alloy in the joints with the quenching and tempering surface layer was much lower than in the joints with the coating (Figure 8). The PAO8 + MoS_2_ used for lubrication also showed a lower wear of the SAE-48 alloy, particularly in the joints with coating. In the joints lubricated with the SAE 5W40 oil, the difference between the wear of the SAE-48 alloy in the joints with the quenching and tempering surface layer and the joints with the coating amounted to approximately 3, 7 and 11 times, respectively, for loads of 10–20 MPa (Figure 8a). Under these lubrication conditions, a proportional increase in friction force was observed with the increase in load on the joints with coating. When lubricated with PAO8 + MoS_2_, a significant reduction in bearing alloy wear was observed in the joints with a coating at a load of 20 MPa: it was lower by half, as compared to wear at 15 MPa (Figure 8b) and approximately three times more than in the joint lubricated with the SAE 5W40 oil (Figure 8).

**Figure 9:**
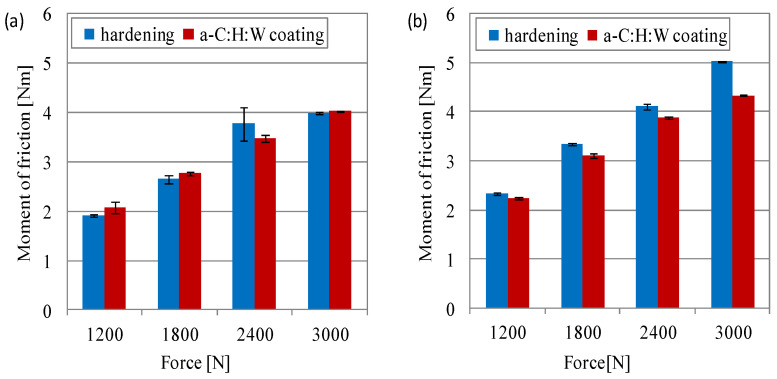
Moment of friction during start-up in joints with non-conformal contact: (**a**) SAE 5W40, (**b**) PAO8 + MoS_2_.

The moment of friction in non-conformal contact during the start-up of the sliding joints showed that lubrication with the 5W50 oil generated lower friction resistance, as compared to the joints in which PAO8 + MoS_2_ was used. In the joints lubricated with the SAE 5W40 oil, the registered moment of friction changed together with the increase in the pressing force. The lower pressing forces (1200, 1800 N) caused a higher moment of friction in joints with a-C:H:W coating than in joints with hardened valve shims. With a pressing force of 2400 N, the moment of friction in the joints with a coating was lower than in the joints with a hardened surface layer. At the greatest pressing force, the moment of friction had the same value for both used counterparts. In the joints lubricated with PAO8 + MoS_2_, it was shown that the increase in the moment of friction was proportional to the increase in the pressing force. The moment of friction was lower in the sliding joints with a coating than in the joints with a hardened surface layer (at 1800 N, the difference was 4%, and at 3000 N, it was 14%) (Figure 9).

**Figure 10:**
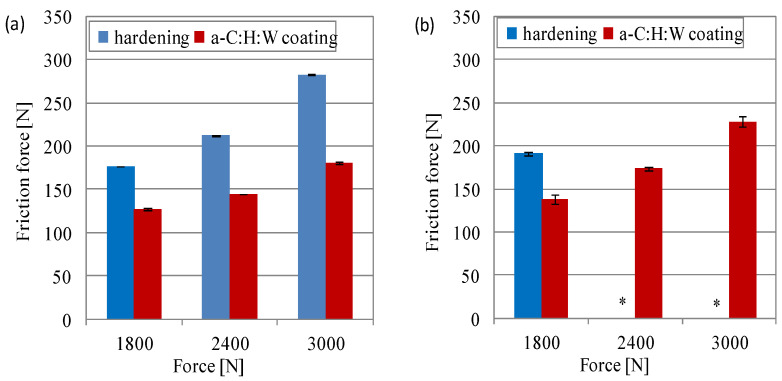
Friction force vs. unit pressure at the end of the test in joints with non-conformal contact lubricated with (**a**) SAE 5W40 and (**b**) PAO8 + MoS_2_ (* seizure of sliding joint).

The friction force in the sliding joints in non-conformal contact with the coating was lower than in the case of joints with a hardened surface layer during lubrication with both oils: SAE 5W40 and PAO8 + MoS_2_. Under these friction conditions, there was a proportional increase in the friction force with the pressing force on the friction areas (Figure 10). Sliding joints lubricated with the SAE 5W40 oil showed a difference in the friction force between the joints with a coating and the joints with a hardened surface layer. These differences amounted to about 28%, 32% and 36%, respectively, at the pressing force of 1800–3000 N (Figure 10a). In the joints lubricated with PAO8 + MoS_2_, the comparison of the tested joints was possible only at the pressing force of 1800 N, because the higher pressing force caused seizure of the joints with a hardened surface layer (Figure 10b). The PAO8 + MoS_2_ oil caused an increase in friction force of approximately 9% (1800 N), 20% (2400 N) and 27% (3000 N), as compared to SAE 5W40 oil in the joint with the coating.

**Figure 11:**
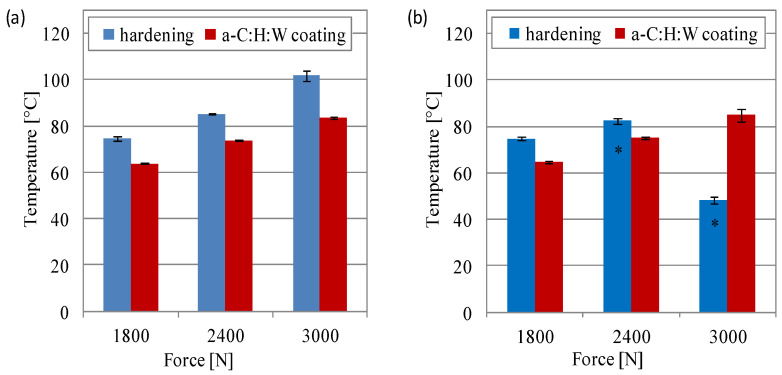
Temperature in contact area vs. unit pressure at the end of the test at joints with non-conformal contact lubricated with (**a**) SAE 5W40 and (**b**) PAO8 + MoS_2_ (* seizure of sliding joint).

The temperature in the contact area showed that the use of a hardened surface layer in the sliding joints caused a greater increase in temperature than in the joints with a-C:H:W coating. On the other hand, the applied lubricant did not show a significant difference in the temperature value in the area of friction. However, when lubricated with SAE 5W40 oil, the difference in temperature between the surface layers tested was a dozen degrees (13–17%); when lubricated with PAO8 + MoS_2_, it was 14% at the lowest force of 1800 N. In the case of greater forces (2400 and 3000 N), seizure occurred, and the recorded temperature was the value achieved at the moment of the maximum recorded friction force on the tribological tester (Figure 11).

**Figure 12:**
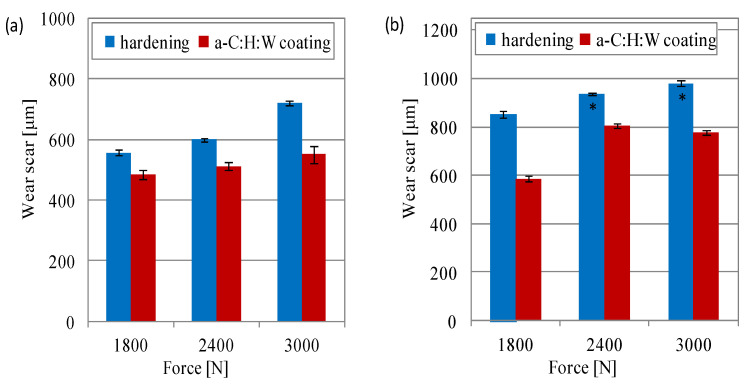
Wear scar on counterpart valve shims lubricated with (**a**) SAE 5W40 and (**b**) PAO8 + MoS_2_ (* seizure of sliding joint).

Measurements of wear scars on the friction surface of valve shims showed similar trends as those observed for the measurements of friction force and temperature in joints in non-conformal contact. In the friction joint lubricated with the SAE 5W40 oil, less wear was observed in the counterparts in joints with a coating in relation to the joints with the commercial valve shims (hardened). Under these lubrication conditions, a proportional increase in the wear value of the counterparts was also observed with the increase in the pressing force, which was applied to both surface layers. The observed difference in the size of the wear scar on the surface with the coating to the one on the hardened surface was within the range of 15 to 24% (Figure 12a). The use of PAO8 + MoS_2_ for lubrication caused an increase in the dimension of the wear scar in relation to the wear scar measured in the joint lubricated with SAE 5W40 oil. Under these lubrication conditions, the joints with the hardened surface layer seized (at the force of 2400–3000 N) and changes in wear in the joint with the coating showed that, at the maximum pressing force (3000 N), the wear scar was smaller than at the pressing force of 2400 N (Figure 12b). The differences in the wear scar between joints with the coating lubricated with SAE 5W40 oil and PAO8 + MoS_2_ were, respectively, 12%, 36% and 29% (Figure 12).

**Figure 13:**
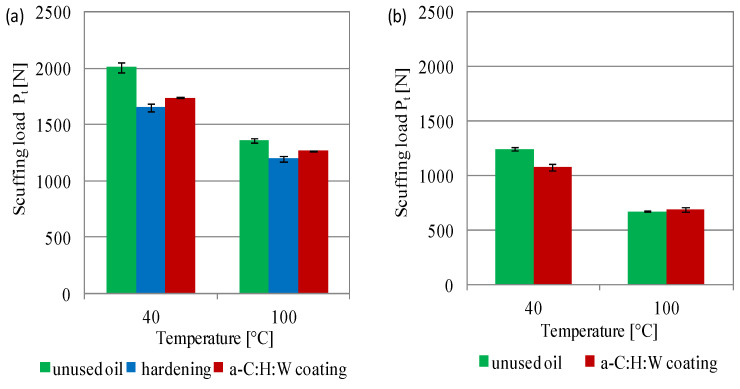
Scuffing load of lubricants: (**a**) SAE 5W40, (**b**) PAO8 + MoS_2_.

**Figure 14:**
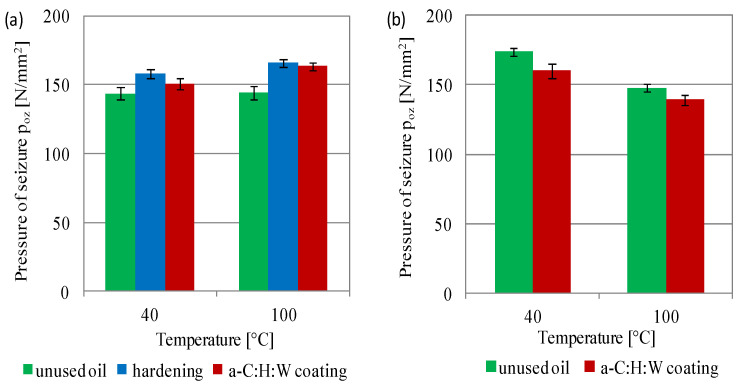
Pressure of seizure of lubricants: (**a**) SAE 5W40, (**b**) PAO8 + MoS_2_.

The tests of lubricants used in the sliding joints with a coating and with a hardened surface layer showed significant differences in oil degradation in non-conformal contact described by scuffing load and pressure of seizure (Figure 13 and Figure 14). The scuffing load for SAE 5W40 oil decreased in the joints with a hardened surface layer, amounting to 18%, and in the joints with a coating it equaled 13%, at a temperature of 40 °C. At 100 °C, the differences were smaller, with the joints with a hardened surface showing 12% scuffing load and the joints with a coating showing 7% load (Figure 13a). An observation of the indicator for the PAO8 + MoS_2_ oil showed that the scuffing load was much lower than that of SAE 5W40 oil, and for the unused oil it was 38% (at 40 °C) and 50% (at 100 °C). The difference in the scuffing load of PAO8 + MoS_2_ between the unused oil and the cooperating oil in a sliding joint with the coating was 11% at 40 °C, and at 100 °C, the difference was at a similar level—about 670 N (Figure 13b). No tests were carried out for PAO8 + MoS_2_ used in sliding joints with a hardened surface layer, due to the fact that the sliding joint was seized and no reliable oil samples were obtained.

The pressure of seizure for SAE 5W40 oil showed an increase in the value with increasing temperature, with the highest values obtained for the oil working in a sliding joint with a hardened surface layer (Figure 14a). In the case of this oil sample, the increase in pressure of seizure was 9% at 40 °C and 15% at 100 °C, as compared to the unused SAE 5W40 oil. As in the case of the scuffing load, no calculations were performed for the PAO8 + MoS_2_ oil due to the scuffing and the lack of oil sample. In the case of the SAE 5W40 oil sample, which worked in the joints with coating, the pressure of seizure increased with increasing temperature by 5% for the temperature of 40 °C and 13% for 100 °C relative to the unused oil. In the case of the PAO8 + MoS_2_ samples obtained from joints with a coating, lower pressure-of-seizure values were obtained compared to the unused oil, amounting to 8% and 5%, respectively (Figure 14b). The seizure pressure decreased with increasing temperature. These differences amounted to 16% for the unused lubricant and 13% for the PAO8 + MoS_2_ used in the joints with coating.

**Figure 15:**
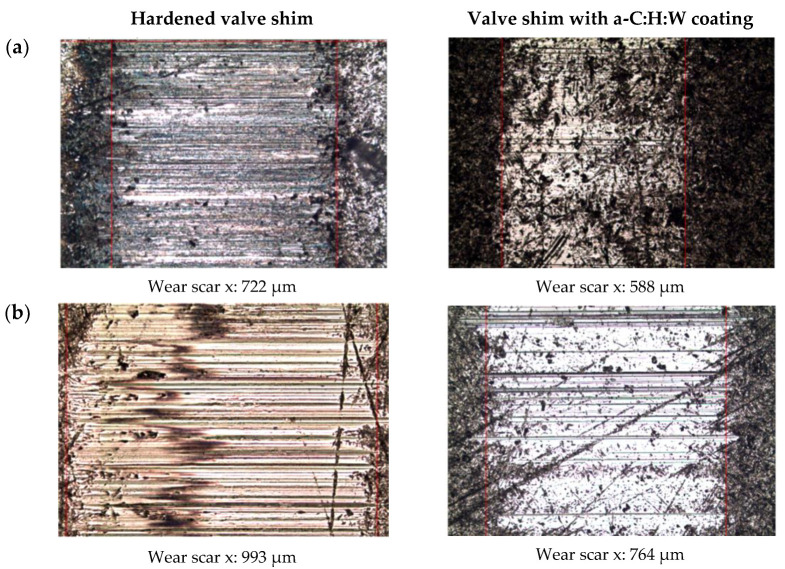
Wear scar on the valve shims after operation in a non-conformal contact (pressing force of 3000 N) lubricated with (**a**) SAE 5W40 and (**b**) PAO8 + MoS_2_ (10× magnification).

Commercial valve shims showed visible signs of wear, including numerous deep scratches. On the other hand, on valve shims with a coating, in the contact area with the ring sample, areas with the original structure of the coating produced in the technological process of its application were observed. The sliding joints with the hardened valve plates seized during lubrication with PAO8 base oil with the addition of molybdenum disulfide, while samples with the a-C:H:W coating showed occasional cracks (Figure 15).

## 4. Discussion

The changes that occurred in the wear processes and parameters of friction in the conformal contact mainly depended on the unit pressures, the microstructure of the surface layer and the chemical reactions between the material elements of sliding joints and the lubricants used [44]. The main wear mechanism observed in the joints was abrasion. The wear of the SAE-48 bearing alloy was caused by the contact of the alloy material with the hard surface layers of the cooperating material (coating) and hard wear products, which, as a result of external forces, led to surface irregularities in these surface layers and more intense abrasive wear of the softer bearing material. Hard wear products also caused such phenomena as chipping and shearing, which may have intensified the wear processes of the surfaces of the friction joints [49,50,51,52]. It is possible that the intensive wear of the SAE-48 was also caused by cutting the damaged areas of the coating with sharp edges (chipping, tearing, scratches) [53].

The a-C:H:W coating used in conformal contact, lubricated with SAE 5W40 commercial oil, generates friction conditions that hinder the creation of stable cooperation conditions. The use of lubrication with PAO8 + MoS_2_ indicates the potential for creating stable cooperation conditions in tandem with the tested coating. This may have been caused by the separation of S and Mo elements, which formed sulfide and molybdenum compounds, and a lubricant was created [37,44,54]. The intensification of the wear processes of the elements of the joint caused an increase in oil contamination, which resulted in a faster degradation of the functional properties of the lubricating oil and a reduction in the durability of the joints lubricated with it [50].

The application of the a-C:H:W coating on working surfaces in non-conformal contact improved the friction conditions in the sliding joints lubricated with the tested oils. A possible mechanism that causes reductions in friction resistance in the joints with the a-C:H:W coating, as compared to the joints with commercial plates, is the phenomenon of coating graphitization [51]. Under conditions of insufficient lubrication, the a-C:H:W coating can take over the role of a lubricant, preventing the occurrence of dry friction and not leading to the damage of the elements of the sliding joints [7,42]. The reduced wear of the a-C:H:W coating may result from the elimination of the material affinity of the steel–steel contact, thus limiting the susceptibility of the friction elements to adhesive wear and the progress of the degradation of the surface layer elements of the sliding joint [28]. The intensity of wear in joints with the a-C:H:W coating may be limited by the presence of a transfer coating, as a result of the transfer of its element from the DLC coating to the cooperating surface. The transfer coating then protects the counterpart material against tribological wear [38].

The tests carried out showed the need for the careful selection of lubricants to cooperate with coatings of the a-C:H:W type in non-conformal contact with a variety of material configurations. The joints lubricated with PAO8 base oil with the addition of MoS_2_ did not protect the joints with hardened valve plates against galling [24]. The operation of friction joints with elements covered with DLC coatings also requires effective protection against the penetration of contaminants into the friction area. The presence of hard impurities may cause coating chipping, thus causing more serious damage to the surface of the mating elements compared to the damage to the elements without carbon coating [39,45].

## 5. Conclusions

The following conclusions can be drawn from this study:

1. The a-C:H:W coating caused an increase in the moment of friction during the start-up of joints with conformal contact compared to the quenching and tempering surface layer, and in the non-conformal contact the coating reduced the moment of friction, as compared to sliding joints with hardened commercial valve plates when lubricated with PAO8 + MoS_2_.
2. In conformal contact, the a-C:H:W coating caused a significant increase in the friction force and decrease in temperature compared to the joints with the quenching and tempering surface layer. In non-conformal contact, the a-C:H:W coating caused decreased friction force and temperature compared to the joints with hardened commercial valve plates.
3. The use of the a-C:H:W coating caused intensive wear of the SAE-48 bearing alloy compared to the quenching and tempering surface layer (lubricants: SAE 5W40 oil—11-fold increase, PAO8 + MoS_2_—46-fold increase). The use of PAO8 + MoS_2_ for lubrication reduced the wear of the SAE-48 alloy by almost three times, as compared to the joints lubricated with SAE 5W40 oil.
4. The wear of the valve shims with the coating was significantly less than the commercial valve shims, and lubrication with the PAO8 + MoS_2_ of the joint with the quenching and tempering surface layer caused its seizure.
5. The determined scuffing load showed that the SAE 5W40 oil has a higher resistance to seizing than PAO8 + MoS_2_ and the processes of exploitation of lubricants led to the deterioration of the scuffing load (for both 40 and 100 °C temperatures).
6. The pressure of seizure for SAE 5W40 oil showed an increase in the value with in-creasing temperature (especially for lubricant exploitation) and decrease for the PAO8 + MoS_2_.
7. The pressure of seizure for SAE 5W40 oil showed an increase with increasing temperature (the highest values obtained for the oil working in the joint with a hardened surface layer). PAO8 + MoS_2_ was characterized by a decreased pressure of seizure with increasing temperature.
8. The use of PAO8 + MoS_2_ base oil for lubrication of sliding joints with the a-C:H:W coating required knowledge of the material composition of the working joints, because under unfavorable lubrication conditions, their seizing might occur (seizure of the sliding joint with hardened valve plate lubricated with the base oil PAO8 + MoS_2_).

## Figures and Tables

**Table 1:** Chemical composition of AISI 4337 (%).

C	Cr	Mn	Mo	Ni	Si	S and P
0.34	1.5	0.65	0.23	1.5	<0.4	<0.035

**Table 2:** Characteristics of SAE 5W40 oil and PAO8 base oil + MoS_2_.

Lubricant	SAE 5W40	PAO8 + MoS_2_
Parameter	Value	Value
Kinematic viscosity at 40 °C [mm^2^/s]	85.3	52
Kinematic viscosity at 100 °C [mm^2^/s]	14.2	8.2
Viscosity index	173	128

## Data Availability

Data are contained within the article.
